# The COVID-19 pandemic posed many dilemmas for policymakers, which sometimes resulted in unprecedented decision-making

**DOI:** 10.1186/s13584-023-00564-x

**Published:** 2023-04-18

**Authors:** Nachman Ash, Noa Triki, Ruth Waitzberg

**Affiliations:** 1 Department of Health Systems Management, Ariel University, Ariel, Israel; 2 Ministry of Health, Jerusalem, Israel; 3 Department of Health Care Management, Faculty of Economics and Management, Technische Universität Berlin, Berlin, Germany; 4 The Smokler Center for Health Policy Research, Myers-JDC-Brookdale Institute, Jerusalem, Israel

**Keywords:** Health policy, Pandemic response, COVID-19 vaccine booster, Decision-making

## Abstract

**Background:**

The COVID-19 pandemic evolved through five phases, beginning with ‘the great threat’, then moving through ‘the emergence of variants', ‘vaccines euphoria’, and ‘the disillusionment’, and culminating in ‘a disease we can live with’. Each phase required a different governance response. With the progress of the pandemic, data were collected, evidence was created, and health technology was developed and disseminated. Policymaking shifted from protecting the population by limiting infections with non-pharmaceutical interventions to controlling the pandemic by prevention of severe disease with vaccines and drugs for those infected. Once the vaccine became available, the state started devolving the responsibility for the individual’s health and behavior.

**Main body:**

Each phase of the pandemic posed new and unique dilemmas for policymakers, which resulted in unprecedented decision-making. Restrictions to individual’s rights such as a lockdown or the ‘Green Pass policy’ were unimaginable before the pandemic. One of the most striking decisions that the Ministry of Health made was approving the third (booster) vaccine dose in Israel, before it was approved by the FDA or any other country. It was possible to make an informed, evidence-based decision due to the availability of reliable and timely data. Transparent communication with the public probably promoted adherence to the booster dose recommendation. The boosters made an important contribution to public health, even though their uptake was less than the uptake for the initial doses. The decision to approve the booster illustrates seven key lessons from the pandemic: health technology is key; leadership is crucial (both political and professional); a single body should coordinate the actions of all stakeholders involved in the response, and these should collaborate closely; policymakers need to engage the public and win their trust and compliance; data are essential to build a suitable response; and nations and international organizations should collaborate in preparing for and responding to pandemics, because viruses travel without borders.

**Conclusion:**

The COVID-19 pandemic posed many dilemmas for policymakers. The lessons learned from the actions taken to deal with them should be incorporated into preparedness for future challenges.

## Background: five phases of the COVID-19 pandemic

The COVID-19 pandemic has evolved through five phases, with different characteristics of the disease, different levels of scientific knowledge and means of treatment, and associated of uncertainty. Each phase also resulted in different governance responses approaches based on these characteristics. In a nutshell, the pandemic evolved as follows:

- “The great threat”—the first and second waves, i.e. from late February to December 2020 [1]. In this phase the new, highly contagious, and fatal virus SARS-CoV-2 was considered to be a great threat. The main responses available from medical and government perspectives were non-pharmacological interventions such as border control, physical distancing and face covering [2]. In Israel and many other countries governance was highly centralized [3, 4]. With few exceptions, governments imposed lockdowns and other physical distancing regulations [1, 5–9].
- "The emergence of variants"—The third wave (December 2020 to February 2021) [10] in Israel was the result of the emergence of the Alpha variant (B.1.1.7) that pointed out the threat of variants that developed rapidly, with higher levels of spread, severity and fatality. Subsequently, other variants developed a strong capacity to evade the immune system, with those infected including people who had recovered and/or been vaccinated.
- “Vaccines euphoria”—The development and approval of vaccines in late December 2020 led people to believe that this was the end of the pandemic. Israel was one of the first countries to launch the vaccination rollout, which was well organized, rapid, and met by a high level of responsiveness from the population [11–13]. Other countries were watching Israel’s pioneer role in vaccinating and learned how to improve their own vaccination policies [14–21]. The public feeling was of having defeated the pandemic with effective vaccines that protect against infection in very high percentages, which was key to the end of the third wave of the disease.
- “The disillusionment”—The surge of the fourth wave during July 2021, caused by the Delta variant surprised many policymakers, as most of the population had been fully vaccinated. Real time data analysis revealed that the effectiveness of the vaccine was waning and the Ministry of Health (MoH) decided to administer a third (booster) dose to the Israeli population. Israel was the world pioneer in rolling out booster doses.

This phase also included the marketing and use of drugs that protected people from severe symptoms and serious illness. These developments changed the attitude of policymakers towards the pandemic. The approach shifted from attempting to prevent infection with non-pharmaceutical interventions, to preventing serious illness with the vaccine and pharmaceuticals. Concomitantly, the state started devolving the responsibility for individuals’ health and behavior and (partially) stepped back from the physical distancing limitations. One of the tools that enabled this relaxation of physical distancing was the establishment of the “Green Pass Policy”, a policy that Israel was also among the first countries to adopt [22].

e. “A disease we can live with”—The upsurge of the Omicron B.1.1.529 variant was fast and the number of people infected was very high due to high infectivity of this variant. The high infectivity made it impossible to avoid widespread infections, both due to the high speed of contagion and to its relative capacity to bypass the vaccine. This phase was also characterized by the attempt to create a “COVID-19 routine”, adapting health systems to cope with this virus along with the many other health needs. Nevertheless, fear and concern persist about the emergence of severe and highly infectious variants.

The analysis of the pandemic through these phases reveals the global strategy of policymakers used in responding to the COVID-19 pandemic. First, policymakers “played defense”, meaning, they tried to protect the population by controlling infections with non-pharmaceutical interventions. Meanwhile, scientists and the industry studied the virus and its threat, and then developed prevention and treatment options such as tests, vaccines and medicines. When those were available, policy shifted gradually to control the pandemic through prevention and treatment, thereby releasing people and the economy from limitations on movement and activity. It is likely that these response phases were not unique to COVID-19 and that they also emerge in many similar types of public health emergencies.

## Main text

### Approving the COVID-19 vaccine third (booster) dose: an example of emergency decision making

During the pandemic, policymakers faced many difficult decisions, which were sometimes posed for the first time. Some of the decisions had to be made under time pressure, insufficient data or evidence, and great uncertainty. Pressing needs sometimes resulted in ‘emergency decision-making’ that resulted in unprecedented decisions. Likewise, in some instances it was difficult to convince politicians, mainly the Prime Minister and the COVID-19 cabinet, to adopt professional recommendations.

One of the most challenging periods in terms of professional policymaking for the MoH, was during the emergence of the 4th wave, the Delta variant wave, between June and August 2021. Israel was ahead of other countries in vaccinating most of the population, and consequently, in overcoming the 3rd (Alpha variant) wave [12]. The lock-down had been lifted on February 7th, 2021, long before the Delta variant emerged. Even the “Green Pass Policy” had been canceled on June 1st 2021,^1^ as the number of COVID-19 cases had been low.

The COVID-19 task force (Magen Israel^2^) detected the Delta variant wave quite early after its emergence in South Africa. The first cases infected with the Delta variant were unvaccinated children, but soon the contagion spread to vaccinated adults. This contagion was unexpected, and raised a great concern and lack of clarity regarding what was going on. As the number of cases escalated, the dilemma regarding whether to re-impose non-pharmacological measures re-emerged. The politicians were reluctant to reinstitute a lock-down or any other restrictions on movement, the education system or businesses. Therefore, it was imperative to understand quickly why the vaccine was not as effective as had been expected. It was unclear whether the Delta variant was bypassing the vaccine or whether the effectiveness of the vaccine was waning.

At this point, the MoH could rely on its detailed data to sort out the source of the problem. Since the MoH had been collecting data centrally from all health providers (hospitals, health plans and clinics) about COVID-19 tests, vaccinations, morbidity and mortality, it was possible to quickly analyze the data and produce evidence that the effectiveness of the vaccine was waning [24].

Immediately, this raised the question of whether to roll out a booster dose of the vaccine. Until that point, the Israeli MoH had approved only vaccines and indications that were previously approved by the FDA. Yet, no epidemiological data regarding the duration of the effectiveness of the vaccine were available from any other country. A booster dose was not being even considered, let alone approved, in other countries. The decision to roll out a booster had to be made in Israel before any other country, and be convincing to the public that it was safe and effective. This was one of the toughest decisions taken by the MoH in view of time limitations. A decision was urgent, as the number of cases was increasing rapidly. There was also a pressing need to develop an evidence-based policy that would be accepted and adopted easily by the public.

Only when the MoH was confident enough about the data analysis and the reason for high infections despite the high vaccination rate, the director general of the MoH could make a decision, accepting the professional recommendation, initially approving the booster vaccine for those older than 60, and subsequently, for younger populations. The booster rollout began with immunocompromised patients on July 13, 2021, and was expanded to people aged over 60 years (on July 30), 50 years (on Aug 12), 40 years (on Aug 19), 30 years (on Aug 24), and the entire population over the age of 12 years on Aug 30 [25].

Yet, the decision to roll out a booster dose alone would not be enough to convince people to get the jab. Not only this decision was unprecedented, the "pandemic fatigue" eroded health workers’ and people’s compliance with public health recommendations. The professional leaders of the MoH together with the minister of health, the prime minister, and the president collaborated to engage with the public and win their trust in the booster dose. The MoH communicated with data that showed that the vaccine efficacy was waning after a period of time, and proved that this was the reason for vaccinated people getting infected with the new virus variant. No less important was to show evidence that a booster dose would be safe and solve the problem of vaccine efficacy waning and subsequent Delta variant wave. It was important for the MoH to show the public that decisions and actions were being taken in wise and careful judgement. Efforts were done to convey the message in an understandable and convincing way to the public. Special attention and reach out was needed to cultural minorities such as the Ultra-orthodox Jews and the Arabs, as vaccine uptake among these groups was lower.

Finally, the data gathered from the booster rollout on its effectiveness and safety was published in the scientific literature, and presented to scientists and governments in other countries, including the FDA authorities, which later approved the booster dose based on Israeli data [24–27].

Despite all these efforts, uptake of the boosters was less than the uptake for the initial doses and less than public health professionals had hoped. Still, the boosters made an important contribution to public health by reducing mortality and morbidity related to COVID-19.

### Looking to the future: What have decision makers learned from the COVID-19 pandemic on how to respond to shocks to the health system?

The broad management of the pandemic in Israel distilled seven key lessons on how to respond to future shocks such as pandemics. The decision-making to roll out the third (booster) dose of the COVID-19 vaccine illustrates these lessons.

First, health technology is key. Every effort should be made to enable researchers, scientists, industries and health workers to develop, produce and disseminate technology such as surveillance and testing material, pharmaceuticals, vaccines and treatment aids as fast as possible. Nations should adopt health technology swiftly based on evidence, and sound health technology assessment. The vaccine was the most cost-effective response to the COVID-19 pandemic [28], and Israel made all efforts to purchase and supply the first, second, and third (booster) doses to its population in a record-time.

Second, leadership is crucial, with important roles for both political and professional leadership. One of the challenges of professional leaders, such as the director of the preventive healthcare services, is to connect the political leaders to science and enable them make evidence-based decisions. It is imperative that politicians understand the rationale of each phase of a shock to the health system, its threats and impacts, as well as the available means to control it. The interaction between professional and political decision makers should be direct and open. This is true in routine times, but it is particularly important during crises. The case of the booster dose was no exception, and one of the advantages of the professional leaders of the MoH was their ability to work hand-in-hand with policy makers and politicians in deciding about the third booster dose, and rolling it out in a timely manner.

Third, a single body should be responsible for orchestrating a prompt and coordinated response. This body should centralize data and action of all stakeholders involved in the response to the pandemic such as ministries and health providers, to name a few. This body should be the focal point for guidelines of action, communication with the public and budgeting response activities. In the case of the booster dose, the MoH took the lead gathering data, making the decision, and coordinating the massive rollout through the health plans.

Fourth, collaboration among the various stakeholders facilitates an effective response. All ministries, providers, and state agencies should work together with the same goal of promoting public health while maintaining other activities unchanged as much as possible. Actors from a broad range of public policy areas such as health, education, security, military, health plans and providers should coordinate action to ensure a coherent response to the pandemic. In the case of the booster rollout, the MoH collaborated with all health providers to collect the data for an evidence-based decision making, and to put into practice the booster vaccination campaign.

Fifth, policymakers need to engage the public and win its trust. Many of the responses to the pandemic depended substantially on people’s behavior such as compliance with non-pharmacological interventions, and then adhering to vaccination guidelines. Trust is built on sincere and transparent dialogue that reflects evidence-based decision making. In situations where evidence is limited, such as the initial phase of a pandemic, policymakers have to be honest and expose the dilemmas and uncertainties involved in decision-making, and justify the rationale behind their decisions. Gaining the public’s trust and adherence to the booster dose was a greater challenge than for previous doses. Direct communication with data and sound explanations was key for the rollout of this third dose.

Sixth, data are essential to understand the dynamics, evolution and effects of the virus or the shock. It is key to build a suitable response, and manage the pandemic. Timely data are valuable, as they reduce uncertainties, guide responses and intervention, and promote public trust. Nations should support central data collection, analysis and open source publication. Data transparency is important in engaging the public. Had the MoH not have the proper and reliable data in a timely manner, the professional decision makers would not be able to make an informed, evidence-based decision to rollout the booster vaccine dose.

Seventh, the pandemic reminded policymakers and the public that all systems are interconnected. Ecosystems, economies and population health around the world influenced one another. Countries understood that viruses (and its variants) travel the world without borders. Policymakers were taught that helping the worse-off (the poor, the sick, the old, to name a few) intra- and extra-borders, is important to protect their own country populations. Scientists and governments understood the importance and need to share knowledge, in a timely and comprehensive manner. Data from the booster campaign shared by Israel was the foundation for other countries to rollout their own booster campaigns.

Operationally, international coordination and cooperation in terms of data collection and sharing and discussing strategic and operational options is essential to control the next pandemic or health shock. Collaboration should be achieved from the onset of a disease with such potential impact, and supporting infrastructure should be established in routine times. International organizations such as the United Nations and the World Health Organization should encourage national leaders to cooperate in preparing for, and responding to, the next pandemic, and to continue to collaborate during its evolution. Threats like the Ebola disease or even Mpox should be tackled at the global level.

Health intelligence units should be established nationally with international cooperation to detect early signs of a threat and share this finding. Continuous surveillance and analysis of local data are essential in every country to detect suspicious patterns that can indicate any early hint of an infectious agent with a potential to disseminate globally.

When a potential pandemic is recognized, all efforts should be put jointly into developing the needed health technologies to prevent and control the pandemic and to disseminate them to all nations. Cooperation of leaders around the globe should encourage policies based on evidence and science.

Coordinated efforts to respond to a pandemic should include tight engagement with the public. Pandemics influence all humans on the globe, healthy and sick, infected or not. Cooperation and compliance of the public with governments' directives is essential to constrain dissemination of the infectious agent.

## Conclusions

All five phases of the COVID-19 pandemic posed many dilemmas for policymakers that had to make many decisions to lead their nation during that challenging period. We described the dilemma that Israel faced during the Delta wave that ended in the decision to administer the booster dose. Leadership, both professional and political, is crucial in handling such situations, especially when engaging the public is crucial. These lessons and others that are described briefly in these paper should be incorporated into preparedness for future challenges.

## Data Availability

Not applicable.

## Abbreviations

MoH: Ministry of Health
FDA: U.S. food and drug administration

## Footnotes

1 https://www.gov.il/he/departments/news/01062021-01.

2 The COVID-19 task force was appointed by the government in mid-July 2020, to take on responsibility for leading the response to the COVID-19 pandemic, including setting short- and long-term health policies to tackle the spread of COVID-19 in Israel. The Task Force was part of the MoH and reported directly to the Prime Minister[23].
